# Induction of Cancer Cell Death by Hyaluronic Acid-Mediated Uptake of Cytochrome C

**DOI:** 10.4172/2157-7439.1000316

**Published:** 2015-10-01

**Authors:** Cindy M. Figueroa, Moraima Morales-Cruz, Bethzaida N. Suárez, Jean C. Fernández, Anna M. Molina, Carmen M. Quiñones, Kai Griebenow

**Affiliations:** 1 Department of Chemistry, University of Puerto Rico, Río Piedras Campus, San Juan, PR 00931, USA; 2 Department of Biology, University of Puerto Rico, Río Piedras Campus, San Juan, PR 00931, USA

**Keywords:** Active targeting, Cytochrome c, Hyaluronic acid, Protein delivery

## Abstract

Effective cancer treatment needs both, passive and active targeting approaches, to achieve highly specific drug delivery to the target cells while avoiding cytotoxicity to normal cells. Protein drugs are useful in this context because they can display excellent specificity and potency. However, their use in therapeutic formulations is limited due to their physical and chemical instability during storage and administration. Polysaccharides have been used to stabilize proteins during formulation and delivery. To accomplish both, stabilization and targeting simultaneously, the apoptosis-inducing protein cytochrome c (Cyt c) was modified with the polysaccharide hyaluronic acid (HA) because its corresponding receptor CD44 is overexpressed in many cancers. Cyt c-HA bioconjugates were formed using low and high molecular weight HA (8 kDa and 1 MDa) with a resultant Cyt c loading percentage of 4%. Circular dichroism and a cell-free caspase assay showed minor structural changes and high bioactivity (more than 80% caspase activation) of Cyt c, respectively, after bioconjugate formation. Two CD44-positive cancer cells lines, HeLa and A549 cells, and two CD44-negative normal cell lines, Huvec and NIH-3T3 cells, were incubated with the samples to assess selectivity and cytotoxicity. After 24 h of incubation with the samples, cancer cell viability was reduced at least 3-fold while CD44-negative control cell lines remained minimally affected. Fluorescence imaging confirmed selective internalization of the Cyt c-HA construct by CD44-positive cancer cell lines. These results demonstrate the development of a drug delivery system that incorporates passive and active targeting which is essential for cancer treatment.

## Graphical Abstract

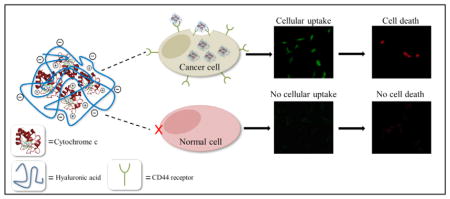

## Introduction

Despite advances in cancer therapy, chemotherapy is frequently still the only option patients with advanced staged cancers have. Many of the available treatment options are plagued by undesired toxic side effects and display a low therapeutic index. Driven by a better understanding of the differences between cancer cells and regular cells, passive and active targeted therapies are being developed to ameliorate the devastating side effects that usually accompany cancer treatment [1]. First, high molecular weight drugs can passively accumulate in tumors by the so-called enhanced permeability and retention (EPR) effect, which is based on the defective vasculature surrounding tumors [2]. Second, covalent modifications (e.g., PEGylation, glycosylation) have increased drug biocompatibility, blood circulation time, and stability [1]. Third, tumor cells frequently over-express transport proteins and receptors, which can be used to enhance the drug tumor specificity by targeting them, for example, by decorating the drug or drug delivery system (DDS) with ligands for the receptors. Such a strategy also allows for the cellular uptake of the cytotoxic drugs which is paramount to their action [1]. If successful, this results in so-called active targeting. Ideally, the development of a drug delivery system should seek to accomplish all three advantages of drug modification mentioned.

Hyaluronic acid (HA) is an attractive candidate for the development of such a DDS because it can be used to construct the bulk matrix, can serve as an active targeting ligand, and could potentially improve drug stability when employing protein drugs like Cyt c used in this work. It is worthwhile to mention that many of the more recent drugs used in cancer treatment are protein drugs, mostly antibodies [3]. HA is a negatively charged, naturally occurring, biocompatible, and biodegradable polysaccharide composed of D-glucuronic acid and N-acetyl-D-glucosamine and is the main component of the extracellular connective tissue [4]. It is involved in wound healing, repair and regeneration of normal cells, as well as the propagation and invasion of cancer cells [5], all of which are regions of high cellular division activity. Polymeric nanoparticles, liposomes, proteins, and anticancer agents have been modified with HA to provide increased circulation half-life [6], biocompatibility [7], amphiphilic character [8], and passive targeting ability [9]. In addition, HA has been used as an active ligand for targeted cancer therapy since its receptor CD44 (cluster of differentiation 44) has been found to be overexpressed in various cancers [10]. It has been observed that HA of high molecular weight (HMW), e.g. 1 × 10^6^ Da, presents high binding affinity towards CD44 and affords subsequent internalization because of the many possible interaction sites with various receptors at once [11–13]. However, smaller HA may still produce the desired effects. Several studies have shown an increase in efficiency of the chemotherapeutic agent (e.g., Mitomycin C, Doxorubicin, and Paclitaxel) and a high antitumor activity towards CD44-positive cells when delivered loaded into HA nano-complexes [14–17]. Paclitaxel, for example, has presented extended blood circulation half-life and reduced liver and spleen accumulation once modified with HA [18].

As already mentioned, many new drugs belong to the group of protein drugs because they offer the advantage of high specificity and therefore low interference with natural biological processes and potency [19,20]. However, proteins are fragile molecules and can undergo denaturation during production, storage, and application. Rapid blood clearance is also a problem in the application of protein drugs [20]. It has been demonstrated that protein stability can be favorably influenced by polysaccharides [21–24]. For example, when conjugated with HA, the blood half-life of insulin was increased and a reduction in enzymatic degradation was observed [25]. Another example is the stabilization of the positively charged tumor necrosis factor (TNF)-related apoptosis-inducing ligand (TRAIL) which displays high anti-cancer potency but a very short blood half-life of less than 30 minutes. Conjugation to HMW HA (2340 kDa) resulted in significant enhancement of the TRAIL stability and pharmacokinetics [26].

Previous studies by us have shown the induction of apoptosis by the protein cytochrome c (Cyt c) once delivered to the cytoplasm of cancer cells [27,28]. Cyt c is found in the mitochondria and is involved in the respiratory process of cells. Apoptotic stimuli trigger Cyt c release into the cytoplasm. Subsequently, programmed cell death is initiated by Cyt c binding to Apaf-1 followed by the caspase cascade activation [29]. Cyt c per se is not a selective drug and will induce apoptosis to any cell when present in the cytoplasm. Thus, in order to avoid damage to normal cells while targeting exclusively cancer cells, we hypothesized that it should be possible to combine the protection and selectivity of HA with the high anticancer potency of Cyt c to construct a powerful DDS. We physically associated Cyt c with two molecular weight HA to evaluate the cytotoxic ability and selective internalization of each system. We also tested the ability of HA to improve protein stability during the association process after exposing Cyt c to detrimental conditions. After proper characterization, each sample was tested in vitro. Two CD44-positive cancer cell lines, HeLa and A549 cells [30–33], and two CD44-negative normal cell lines, Huvec and NIH-3T3 cells [34–36], were incubated with the samples to evaluate cellular internalization and cytotoxicity.

## Materials and Methods

Cytochrome c from equine heart and hyaluronic acid sodium salt (1.8 × 10^6^ Da) were from Sigma-Aldrich (St. Louis, MO). Sodium hyaluronate, 8293 Da, was purchased from Lifecore Biomedical and hyaluronic acid-NH_2_ (HA-NH_2_, 10 kDa) from Creative PEGWorks (Winston-Salem, NC). HeLa (human cervix adenocarcinoma), A549 (human lung adenocarcinoma) (ATCC^®^ CCL185^™^), HUV-EC-C [HUVEC] (human umbilical vein endothelial cells) (ATCC^®^ CRL-1730^™^), and NIH-3T3 (mouse embryo fibroblast) (ATCC^®^ CRL1658^™^) cells were from the American Type Culture Collection (Manassas, VA). 4′, 6-Diamidino-2-phenylindole (DAPI), propidium iodide (PI), fluorescein-5-isothiocyanate (FITC), and ProLong^®^ gold antifade reagent were purchased from Invitrogen (Carlsbad, CA). All other chemicals (reagent or analytical grade) were purchased from various suppliers and used without further purification.

### Cyt c-HA bioconjugate preparation

**Figure 1:**
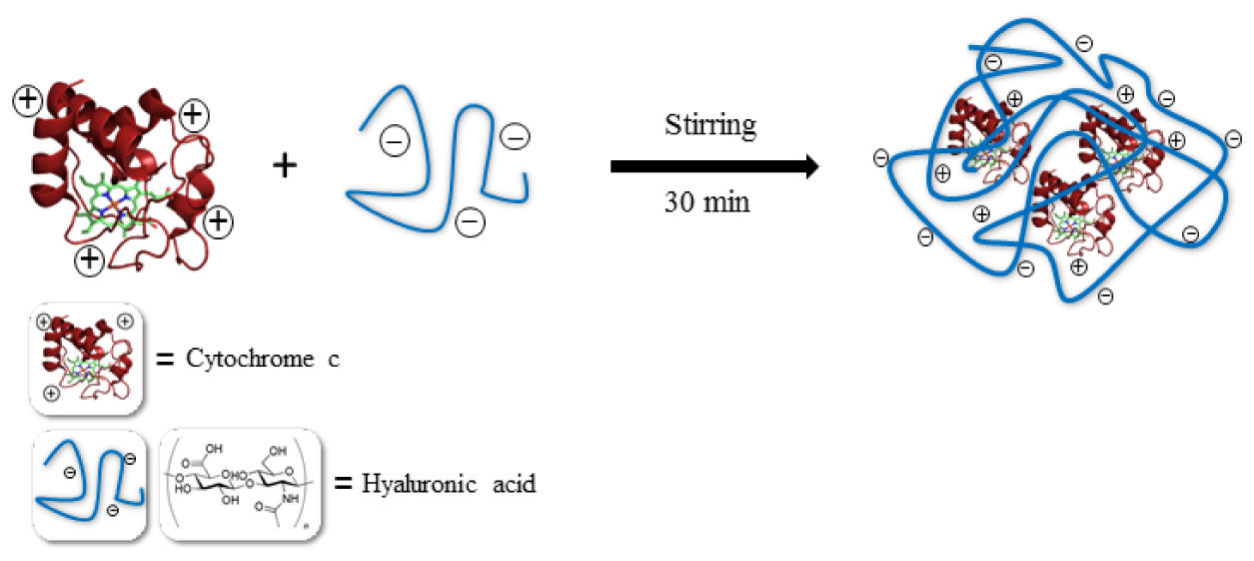
Scheme of Cyt c-HA bioconjugate formation.

Cyt c-HA bioconjugates were formed through physical adsorption. Briefly, native Cyt c and HA (8293 Da and 1.8 × 10^6^ Da) (Figure 1) were dissolved separately in 10 mM PBS, NaCl 150 mM pH 7. Cyt c was dissolved at a concentration of 4 mg/ml and was then added drop-wise to previously dissolved HA. Different concentrations of HA were used to change the DDS loading. After addition, the solution was stirred for 15 min followed by sonication for 30 min and the product was lyophilized.

### Cyt c-HA bioconjugate characterization

#### Protein loading

The Cyt c loading was obtained from its absorbance at 408 nm (ε:6.7 ml/mg·cm^−1^) (Table 1). Around 1 mg of the lyophilized sample was dissolved in nanopure water and its absorbance measured at 408 nm measured and the Cyt c concentration calculated. Subtraction of this amount from the total weight of the system produced the amount of HA in the system. These values were used to calculate the Cyt c loading percentage in every sample; Mean ± SD (n=3).

#### Dynamic light scattering and zeta potential measurements

Formation of bioconjugates was achieved through ionic interactions between the positively charged Cyt c and the negatively charged HA. Dynamic light scattering was used to obtain size and zeta potential values of bioconjugates after formation. Data was collected in a Zetasizer Nanoseries from Malvern using polystyrene disposable cuvettes for size measurements and folded capillary disposable cuvettes for zeta potential measurements. Samples were dissolved in 0.1M PBS and 150 mM NaCl at pH 7. Size values are the average of 15 scans. Data of zeta potential values was collected from 3 runs and each was the average of 15 measurements.

#### Circular dichroism (CD) spectroscopy

To observe changes in Cyt c structure after DDS construction, CD spectra were obtained for each sample. Cyt c and Cyt c-HA bioconjugates were dissolved in 10 mM PBS at pH 7.4 to obtain a final Cyt c concentration of 0.09 mg/ml. CD spectra were obtained using a JASCO J-1500 High Performance CD spectrometer at room temperature. Near-UV spectra were measured from 260–350 nm to observe the protein tertiary structure and the Soret region (heme group) was measured from 380–450 nm using a 10 mm quartz cuvette. Each sample was scanned twice to obtain an averaged spectrum from which the background of a nanopure water blank was subtracted.

#### Cell-free caspase 9 activation assay

A cell-free caspase 9 activation assay was used to observe changes in caspase activation. The cell lysate was obtained as described by us [27]. Briefly, after regular passage, the cells were washed thrice with medium, Hepes buffer, and cell-free assay buffer followed by three freeze-thaw cycles. Cells were centrifuged at 10,000 rpm to remove mitochondria and avoid effects from endogenous Cyt c. The cell-free reaction was initiated by adding Cyt c or the different Cyt c-HA formulations (e.g. 100 μg/mL of Cyt c-HA) to freshly purified cytosol (3 mg/ml) in a total reaction volume of 50 μL. The reactions were incubated at 37°C for 150 min. Afterwards, the caspase 9 assay was performed following the manufacturer’s protocol (CaspACE^™^ assay; Promega, Madison, WI). The plate was incubated overnight at room temperature and the absorbance in each well was measured at 410 nm using a Thermo Scientific Multiskan FC. Cyt c was used as positive control and α-lactalbumin (no apoptotic protein) as negative control. All measurements are the average of at least three replicates.

### Cell culture experiments

HeLa, A549, HUVEC, NIH-3T3 cells were maintained in accordance with their respective ATCC protocols. Briefly, HeLa and A549 cells were cultured in minimum essential medium (MEM), NIH-3T3 cells in Dulbecco’s Modified Eagle’s Medium (DMEM), and HUVEC cells in F-12K medium, 0.1 mg/ml heparin, and 0.03–0.05 mg/ml endothelial cell growth supplement. All media contained 1% L-glutamine, 10% fetal bovine serum (FBS), and 1% penicillin. All cell lines were kept in a humidified incubator under 5% CO_2_ and 95% air at 37°C. Experiments were conducted before cells reached 25 passages. For cell viability and confocal microscopy experiments, cells were seeded in 96-well plates or chambered cover-slides (4 wells), respectively, for 24 h. Subsequently, cell growth was arrested by decreasing the FBS concentration in the medium to 1% for 18 h. Then, cells were incubated with the different Cyt c-HA bioconjugates and controls for either 6 or 24 h.

#### Cell viability assays

Cell proliferation was measured using the CellTiter 96 aqueous non-radioactive cell proliferation assay from Promega Corporation after 6 or 24 h of sample incubation, as indicated. All cell lines were incubated with various concentrations of Cyt c (0.15, 0.075, 0.0375, 0.01 mg/ml). Negative (HA and Cyt c) and positive (2 μM staurosporine) controls were also tested. After incubation, 20 μl of 3-(4, 5-dimethylthiazol-2-yl)-5-(3-carboxymethoxyphenyl)-2-(4-sulfophenyl)-2H-tetrazolium, inner salt (MTS) were added to each well. Plates were left for 1 h at 37°C under 5% CO_2_ atmosphere and then the absorbance at 492 nm was measured using a microplate reader. Each data point is at least an average of 8 measurements.

#### Bioconjugate stability

To observe the bioconjugate stability, samples were dissolved in 1 ml of 10% FBS supplemented MEM to a final concentration of 0.15 mg/ml of Cyt c. Once dissolved, they were sonicated for 30 min following incubation at room temperature for various times (i.e., 0, 1 h, 6 h, 24 h, 48 h, and 120 h) using a Bransonic 3510 ultrasonic cleaner. Afterwards, samples were incubated with the HeLa cells and the cell viability was measured as described above.

#### Visualization of cell death with fluorescent probes upon Cyt c-HA bioconjugates incubation

Confocal microscopy was used to visualize and confirm cell death after incubation with the Cyt c samples using DAPI/PI staining in A549 cells as described by us [28]. In brief, cells were seeded in a 4-chambered slide system and incubated with controls (HA and Cyt c) and Cyt c-HA bioconjugates at 0.03 mg/ml Cyt c concentration at 37°C for 24 h. Cells were washed with PBS and incubated with DAPI which stains the nucleus blue, and PI that stains DNA red in case of nuclear membrane permeability and chromatin condensation because of cell death, for five minutes each. Cells were then fixated with 3.7% formaldehyde for 30 min and washed thrice with PBS. To avoid photobleaching of the fluorescent dyes we added 100 μl of ProLong^®^ to each well. The slides were then covered with coverslips. If slides were not observed immediately they were left overnight at room temperature and then at 4°C until visualization under the microscope. After incubation, the chambered plates were observed under a Zeiss laser-scanning microscope 510, using a 40X objective to assess co-localization of DAPI and PI in cells. The excitation for DAPI was at 405 nm and for PI it was 561 nm. The emission of DAPI was observed at 420–480 nm and of PI at 609–684 nm.

#### Cellular uptake of Cyt c-HA bioconjugates

Controls (HA and Cyt c) and Cyt c-HA bioconjugates were reacted with FITC to allow observing their internalization. A549 cells were incubated with HA-NH_2_-FITC, Cyt c-FITC, and bioconjugates (Cyt c concentration of 0.02 mg/ml) at 37°C for 24 h. After removing the media, chambers were washed thrice with PBS following fixation with formaldehyde and washing with PBS. Instead of using Prolong^®^, we used glycerol to avoid interference with FITC and photobleaching. Slides were covered and left overnight at room temperature and observed by confocal microscopy using the Zeiss 510 mentioned before with a 40X objective. FITC was excited at 488 nm and observed at 505 nm.

Cellular uptake differences among cell lines were also observed using confocal microscopy. HeLa, A549, and NIH-3T3 cells were seeded in chambered cover-slides as previously mentioned. Cells were incubated with bioconjugates at a Cyt c concentration of 0.02 mg/ml for 30 min, 2 h, and 6 h. After incubation, cells were prepared and observed as mentioned above.

#### Statistical analysis

All experiments were carried out at least in triplicate unless specified otherwise. Values are mean ± SD. The Mann-Whitney U Rank Sum test was performed using the STATA^®^ 11.0 software to determine statistically significant differences (p <0.05) between controls and samples in biostability, cell viability, and cellular uptake experiments.

## Results and Discussion

### Physical characterization of Cyt c-HA bioconjugates

#### Protein loading

One of the general ideas of this work was that Cyt c should potentially accumulate in tumors by the EPR effect after successful coupling to HA [37]. It is important to emphasize that smaller molecules (i.e., <10 kDa) diffuse faster and deeper than larger molecules (i.e., 2000 kDa) into cancer tissues. However, they also rapidly diffuse back into the vasculature and are not tissue selective [1]. The proper molecular weight of the drug delivery system is crucial to achieve selective accumulation in tumor tissues by passive targeting driven by the enhanced permeability and retention (EPR) effect. Two HA preparations with very different molecular weights (8 kDa and 1 MDa) were used in the construction of Cyt c-HA bioconjugates to evaluate their comparative efficacy to deliver Cyt c to cancer cells.

Differences between samples synthesized with either HA were assessed through physical and *in vitro* characterization. In general, a high drug loading is beneficial to deliver a potent system [38], but it was unclear how this would influence cellular uptake. To assess the effect of drug loading on cellular uptake and other critical parameters, we consequently produced bioconjugates with different protein loading by using various synthesis conditions (see Methods for details). The protein loading measured for the bioconjugates obtained were 35 ± 5% and 4 ± 1% for Cyt c-HA 8kDa and 16 ± 1% and 4.3 ± 0.8% for Cyt c-HA 1MDa, respectively. Throughout the manuscript, bioconjugates with the higher protein loading were labeled with a^★^ at the end. All results from physical characterization are summarized in Table 1.

#### Zeta potential values

Zeta potential values were used to confirm the physical interaction between Cyt c and HA. Since the interaction of HA and Cyt c is based on charge-charge attraction, measurement of the zeta potential is very useful to follow the complex formation [26]. We observed the same pattern of changes as for the Cyt c zeta potential before and after bioconjugate formation [26] (Table 1). Zeta potential values are also relevant to the aggregation propensities of the system. If the zeta potential is low (between +30 and −30 mV) then the dispersion is considered unstable and the particles will eventually aggregate; higher values are characteristic of stable dispersions or emulsions. One has to keep in mind that this is a rough assumption and that aggregation tendencies can vary depending on particle size, pH, among other parameters [39]. In our case, lower loading and larger HA MW produced lower zeta potential values (Table 1). Even though our complexes might undergo aggregation since we did not achieve −30 or +30 mV of zeta potential, the bioconjugate stability in vitro was assessed to confirm bioactivity and cytotoxicity.

#### CD spectroscopy

**Figure 2:**
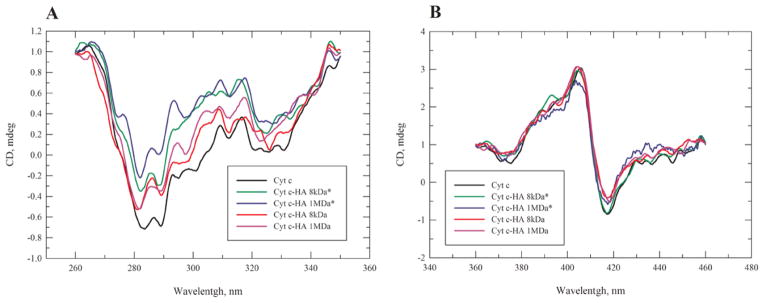
(A) CD spectra of Cyt c (black line), Cyt c-HA 8kDa* (green line), Cyt c-HA 1MDa* (blue line), Cyt c-HA 8kDa (red line), and Cyt c-HA 1MDa (violet line) in the near-UV and (B) Heme band region.

Changes in the Cyt c environment by the conjugation to HA could affect its tertiary structure including the heme binding pocket. Tertiary structural changes were characterized by CD spectroscopy in the near-UV region (260–340 nm) and the Soret region (360–450 nm), respectively (Figure 2).

The CD spectra show moderate changes in the near UV region (260–340 nm) of the bioconjugates compared to Cyt c (Figure 2A). A reduction in CD intensity indicates some loss in tertiary structure, presumably due to the interactions between Cyt c and HA. Results are in accordance with previous research that showed changes in the Cyt c tertiary structure once linked to negatively charged species [40]. Cyt c-HA complexes with lower Cyt c loading showed a less pronounced spectral change than those with higher Cyt c loading. No significant changes were observed in the Soret region (360–450 nm) of the bioconjugates when compared to Cyt c (Figure 2B). This region is characteristic of the environment surrounding the protein heme group, close to the binding epitope of Cyt c with Apaf-1. The CD spectrum shows a pronounced Cotton effect [21], which is quite sensitive to structural perturbations in the heme binding pocket [27,41]. In summary, we conclude that Cyt c tertiary structure might be affected to some extent by the complex formation but the heme group environment remained largely unaltered.

#### Cell-free caspase-9 activation assay

Cell-free caspase-9 activation assay was used to assess the biological activity of Cyt c after complex preparation. In order to induce apoptosis, Cyt c must interact with Apaf-1 after its release from the mitochondria [42]. This interaction leads to the formation of the apoptosome, which subsequently activates the caspase cascade. We selected caspase-9 because it is part of the Cyt c-mediated caspase cascade pathway. All samples were compared to the caspase activation produced by commercially obtained Cyt c before complex formation. Cyt c-HA bioconjugates were able to activate caspase-9 practically as well as the Cyt c control (Table 1). It is apparent that the Apaf-1 interaction site was not affected by the HA association. This agrees with CD results that showed no structural alteration of the heme group and heme-binding pocket.

### Cell culture experiments

Once confirmed by the cell-free assay that the Cyt c-HA bioconjugates had the capability to interact with Apaf-1 and induce apoptosis, we investigated the effect of the different bioconjugate formulations on cancer cells using cell lines that overexpressed the CD44 receptor and those that did not as control.

#### Cell viability assays

**Figure 3:**
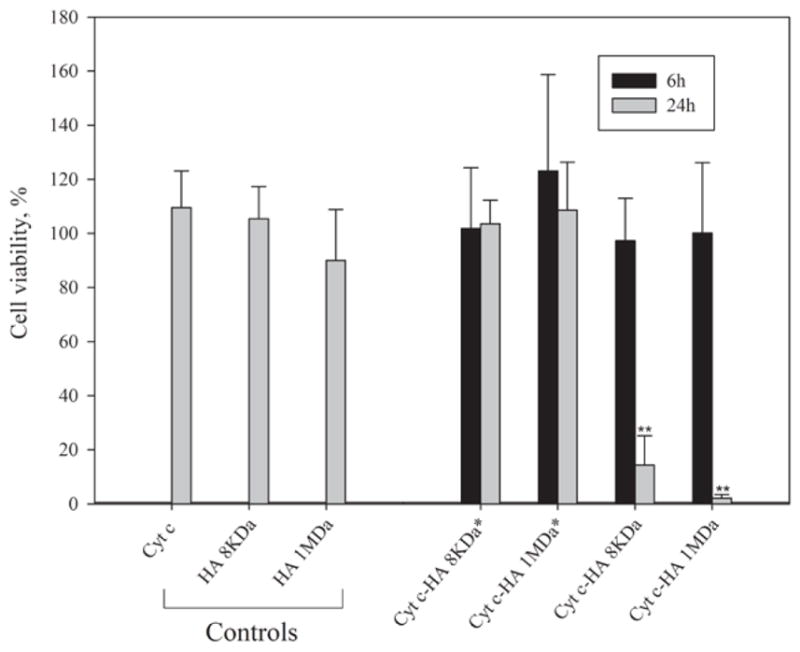
HeLa cell viability of after 6 and 24 h of incubation with bioconjugates and controls. HeLa cells were incubated with bioconjugates (0.15 mg/ml of Cyt c) for 6 h (black columns) and 24 h (gray columns). Asterisks (**) indicate a statistically significant difference between samples after 6 h and 24 h of incubation, p<0.001. Controls are the individual elements employed at the highest experimental concentration in the experiments with the conjugates.

Before incubating cells with the Cyt c-HA bioconjugates, we evaluated Cyt c toxicity by performing a dose-response experiment. At a concentration of >0.15 mg/ml, Cyt c induced some cell death in both, HeLa and A549 cells (Figure 1). Therefore, experiments to measure cell viability after incubating cells with Cyt c-HA bioconjugates were carried out using a Cyt c concentration of less than 0.15 mg/ml. In addition, cell viability was measured at different incubation times (6 h and 24 h). Surprisingly, our results show that when HeLa cells were incubated with the bioconjugates having high Cyt c loading, i.e. Cyt c-HA 8kDa^★^ and Cyt c-HA 1MDa^★^, no cytotoxicity was observed after either 6 h or 24 h of incubation (Figure 3). In contrast, both bioconjugates with lower Cyt c loading (Cyt c-HA 8 kDa and Cyt c-HA 1MDa) were able to induce a substantial reduction in cell viability to 14% and 2%, respectively, after 24 h of incubation. As controls, HA 8 kDa and HA 1 MDa were added to HeLa cells at the highest concentration used in the corresponding experiments with the conjugates. No significant cytotoxicity was observed after 6 h or 24 h. Therefore, Cyt c-HA 8kDa and Cyt c-HA 1MDa were the samples selected to be used in further experiments.

Interestingly, somewhat contrary to our initial expectation, results show that lower Cyt c loading of the bioconjugates produced a larger reduction in cell viability. However, similar results were published by Mero et al. where HA-insulin conjugates with the lowest protein loading caused the largest effect on blood glucose level. They hypothesized that steric entanglement of insulin could be responsible for this [25]. In our case, a possible explanation could be increasing inefficiency of HA to bind to CD44 at increasing Cyt c loading. Too much Cyt c in the bioconjugate might reduce the effective interaction between HA and CD44, thereby depleting cellular uptake. Therefore, a higher amount of HA, which is the active ligand against CD44, permits the entrance of the cytotoxic drug to produce cell death.

#### Bioconjugate stability

**Figure 4:**
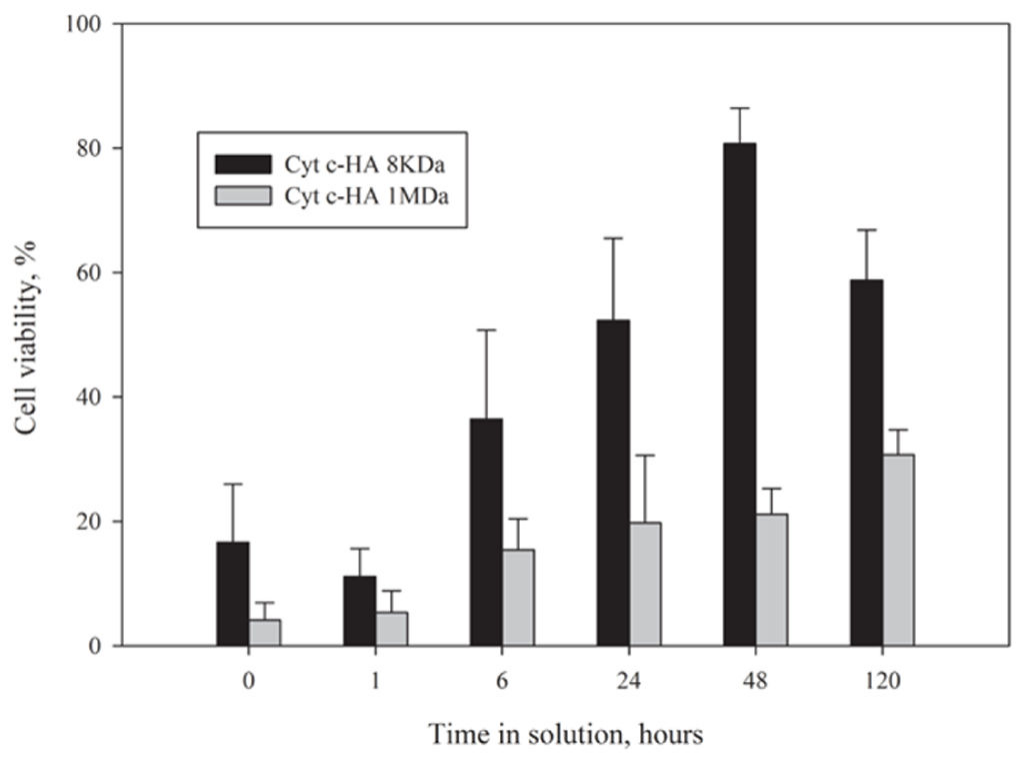
Liquid-state stability of Cyt c-HA bioconjugates. Samples were dissolved in culture media and incubated at room temperature for various times. HeLa cells were incubated for 24 h with the bioconjugates and cell viability was measured.

Since zeta potentials seem to indicate low stability for both Cyt c-HA 8kDa and Cyt c-HA 1MDa (−11.9 and −22 mV, respectively, (Table 1) we tested their liquid-state stability by measuring their effect on cell viability after incubation in solution. The bioconjugates were dissolved in cultured media and kept at 25°C for various times. HeLa cells were then incubated with the samples to assess changes in their cytotoxic effect (Figure 4).

Cell viability results were in accordance with the prediction of the zeta potential values. The bioconjugate with higher zeta potential presented a higher cellular cytotoxic effect even after being in solution for 48 h. On the other hand, after 48 h in solution, Cyt c-HA 8kDa showed a significant increase in cell viability (more than 80%). Even though, zeta potential values indicated that our bioconjugates should show a low stability in solution, it was clear that at least Cyt c-HA1MDa can still produce significant cytotoxicity even after 120 h in solution (31 ± 4%). Cyt c-HA 8kDa only produced significant cell death for few hours.

To assess the specificity of the Cytotoxicity of Cyt-HA 8kDa and Cyt c-HA 1MDa, we tested their cytotoxic effect on various cell lines. HeLa and A549 cells were used as cancer CD44-positive cell lines while NIH-3T3 and Huvec cells were used as normal CD44-negative cell lines. For these experiments, cells were incubated with the bioconjugates for 24 h to achieve the maximum effect on cell viability.

**Figure 5:**
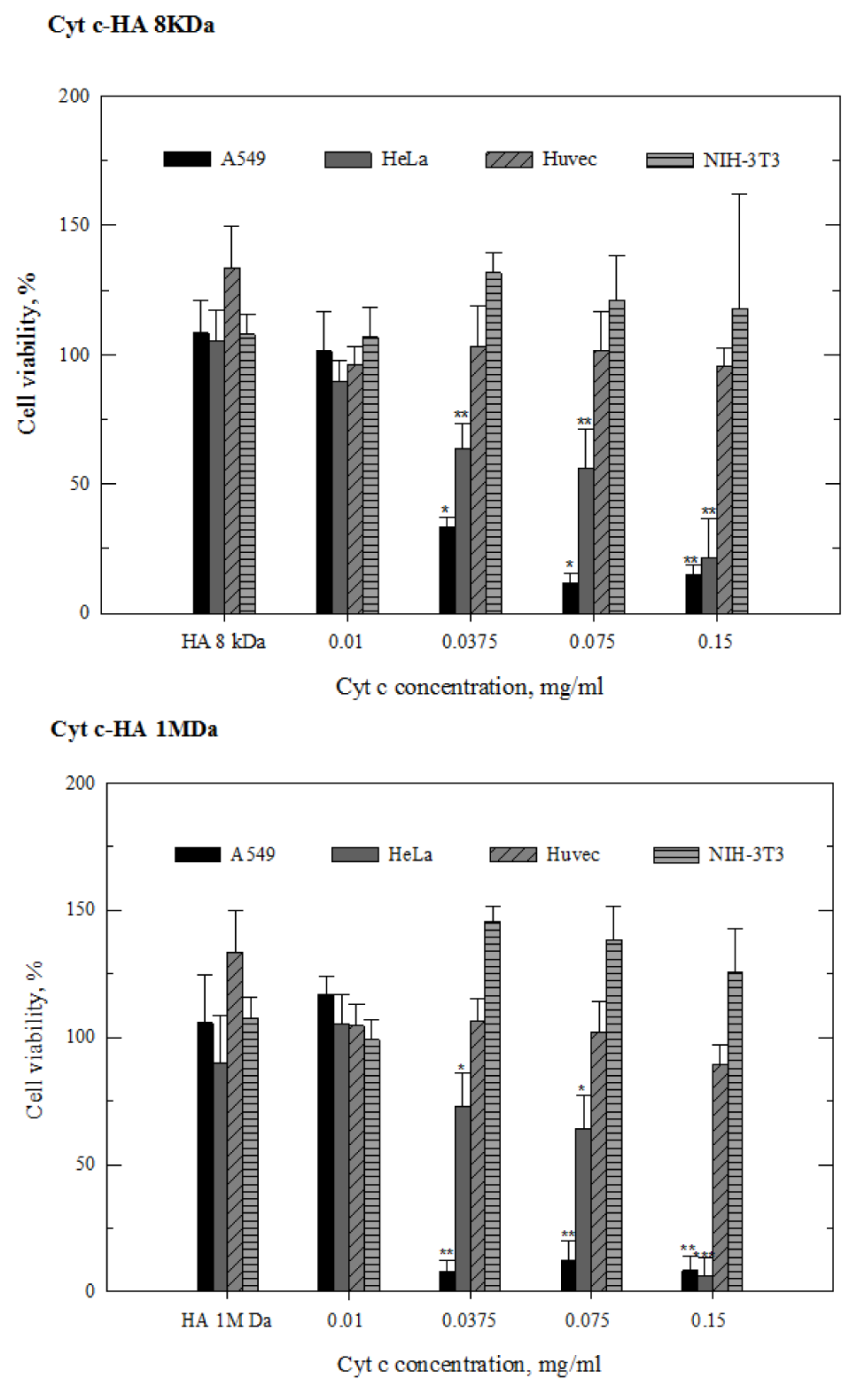
Viability of HeLa, A549, Huvec, and NIH-3T3 cells after incubation with different concentrations of Cyt c-HA 8kDa and Cyt c-HA 1MDa. Cells were incubated for 24 h with Cyt c-HA 8kDa (upper panel) and Cyt c-HA 1MDa (lower panel). Asterisks (*) indicate statistically significant difference from the control (HA) in corresponding cell line, p<0.05, ** p<0.001, and *** p<0.0001.

It was evident that Cyt c-HA bioconjugates had no cytotoxic effect on normal CD44-negative cell lines (Huvec and NIH-3T3 cells) while in CD44-positive cell lines (HeLa and A549 cells) the effect was substantial (Figure 5). Of the CD44 positive cell lines, A549 cells were more susceptible to cytotoxic effects induced by both, Cyt c-HA 8kDa and Cyt c-HA 1MDa, than HeLa cells. As desired, both Cyt c-HA bioconjugates were able to reduce cell viability in CD44-positive cancer cell lines whereas no significant cytotoxicity was observed with either CD44-negative normal cell lines over the concentration range tested. These results concur with the idea that only the cells overexpressing CD44 were able to uptake the HA-Cyt c conjugates.

#### Cyt c-HA bioconjugate uptake and cell death

**Figure 6:**
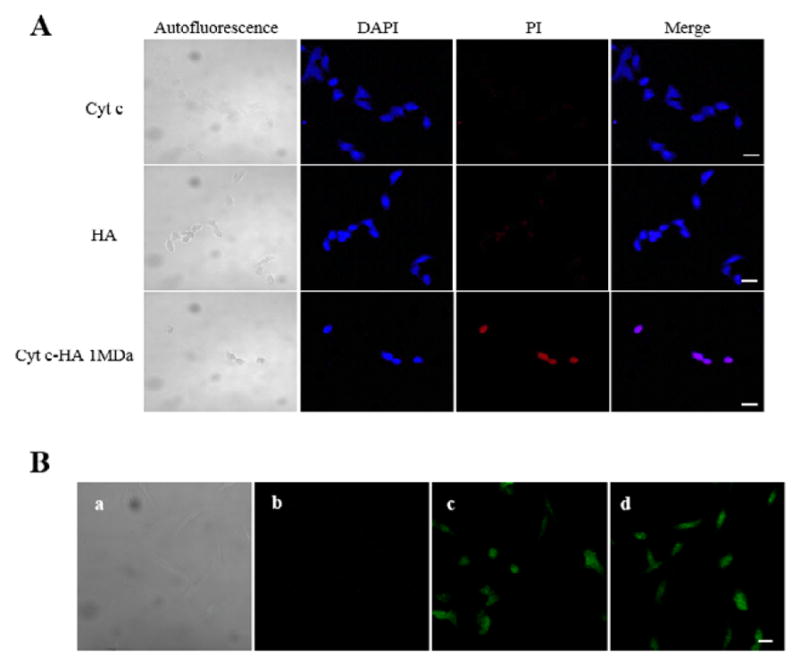
DAPI/PI staining and cellular uptake in A549 cells after 24 h of incubation. A) Co-localization of DAPI (blue) and PI (red) indicate cell death. Controls (Cyt c, first row, and HA, second row) do not cause cell death while Cyt c-HA 1MDa caused significant cell death. B) Cellular uptake of Cyt c (b), HA (c), and Cyt c-HA 1MDa (d). Autoflourescence of A549 cells is observed in a. Bar scale=20 μm.

To further confirm that the effect on cell viability was due to apoptosis induced by the Cyt c-HA delivery into the cytoplasm, a study of DAPI and PI co-localization was conducted using confocal laser scanning microscopy. DAPI stains the cellular nucleus while PI stains condensed and fragmented chromatin relying on nuclear fragmentation [28]. Therefore, co-localization of DAPI and PI seen after cell incubation with Cyt c-HA 1MDa (Figure 6A) confirms cells undergoing cell death. The lack of co-localization in cells incubated with either Cyt c or HA alone demonstrates the lack of toxicity when using the components isolated. This result establishes the synergistic action of Cyt c and HA when they are combined into one conjugate system.

To evaluate that cell death was due to cellular uptake via the CD44 receptor mediated endocytosis, internalization of Cyt c-HA and its individual components was tested by confocal laser scanning microscopy (Figure 6). Cyt c, HA 1MDa and Cyt c-HA 1MDa were modified with FITC to observe their localization within cells.

As expected, no signal of FITC-labeled Cyt c was observed inside the cells since Cyt c cannot penetrate the cytoplasmic membrane (Figure 6b). FITC-labeled HA, in contrast, was able to enter the cells clearly visualized inside the cells (Figure 6c). Importantly, HA also allowed Cyt c uptake into the cells, as the results with FITC-labeled Cyt c-HA 1MDa demonstrate (Figure 6d). Thus, the interaction of HA with the cells, most likely through its receptor CD44, allows the internalization of the system. This fact makes it a suitable candidate as an anticancer therapeutic.

#### Specificity of Cyt c-HA bioconjugates

**Figure 7:**
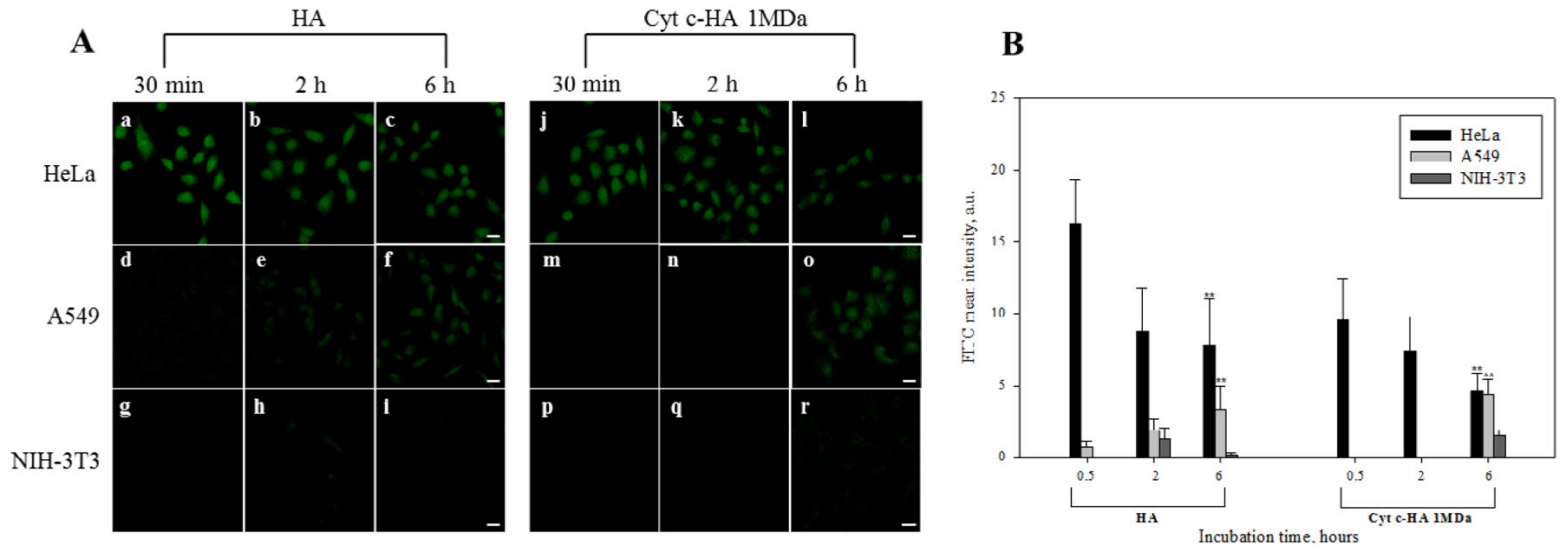
Cellular uptake of FITC-HA and FITC-Cyt c-HA 1MDa by various cell lines. A) Confocal images of cancer (HeLa and A549 cells) and normal (NIH-3T3) cell lines visualized after 30 min, 2 h, and 6 h of incubation. Left panel=HA, Right panel=Cyt c-HA 1MDa. Bar scale=20 μm. B) Quantitative FITC intensities from A. Cells from each image were randomly selected to calculate mean values ± SD. Asterisks (**p<0.001) represent statistically significant difference between CD44-positive cells to control CD44-negative cells (NIH3T3).

After detection of Cyt c-HA 1MDa cell-line specific cytotoxicity in cell viability assays, we explored differences in the bioconjugate internalization using CD44 over-expressing cancer cell lines (HeLa and A549) and a normal cell line not over-expressing the receptor (NIH-3T3). Many physicochemical properties can affect the DDS cellular internalization, such as, ligand orientation, charge, and density [1]. For this reason, we modified the bioconjugates with a fluorescent probe to visualize its internalization. Confocal laser scanning microscopy revealed interesting facts about Cyt c-HA 1MDa internalization among the cell lines treated. HeLa cells incubation with either HA or Cyt c-HA 1MDa showed rapid sample internalization after 30 min of incubation (Figure 7). Surprisingly, the FITC-labeled bioconjugate did not show an increase in fluorescence over time (Figures 7b,c,k and l). This can suggest saturation of CD44 receptors present on HeLa cells. This type of saturation has been observed in macrophages due to the slow CD44 representation [43]. In both cancer cell lines, Cyt c-HA 1MDa showed a lower internalization rate compared to HA. Interactions between HA and Cyt c can decrease the availability of HA to interact with CD44 thus lowering its uptake.

Another finding is the difference of sample internalization between HeLa and A549 cells. Since according to cell viability assays, A549 cells were more susceptible to the cytotoxic effect of the bioconjugates (Figure 5), we expected to see higher and or faster internalization of both, HA and Cyt c HA 1MDa, compared to HeLa cells (Figure 7). However, results show the contrary: HeLa cells exhibited showed an enhanced fluorescence in their cytoplasm after 6 h of incubation with FITC-HA and FITC-Cyt c HA 1MDa compared to A549 cells. Differences in the level of receptor overexpression among cell lines could explain this unexpected result [32]. For example, HA can interact with cell surface receptors other than CD44, such as RHAMM (receptor for hyaluronan-mediated motility, CD168), HARE (HA receptor for endocytosis), ICAM-1 (intracellular adhesion molecule-1), and LYVE-1 (lymphatic vessel endocytic receptor) [10]. Thus, interactions with receptors other than CD44 can also increase cellular uptake. Also, high cellular internalization might not mean high cell death. For example, up-regulation of anti-apoptotic pathways, such as, Erk1/2 and Akt can deplete the cytotoxic ability of the delivered drug [44]. Many alterations in different cancer cell lines can produce drug resistance avoiding the desired drug cytotoxic effect. Many upregulated genes related to anti apoptotic pathways have been found in HeLa cells [45], which could explain the low cytotoxicity observed in HeLa cells compared to A549 cells.

As anticipated, little internalization of FITC-HA and FITC-Cyt c-HA 1MDa was observed in normal CD44 negative cells (NIH-3T3 cells) (Figures 7g,h,i,p,q and r). Results therefore confirm that Cyt c-HA 1MDa is a CD44-positive cancer cell specific drug delivery system.

## Conclusions

In this study, we have presented evidence of the potential of Cyt c-HA bioconjugates as targeted anticancer therapeutics. We demonstrate that the bioconjugates were (i) properly constructed, (ii) selectively internalized in CD44 over expressing cancer cells, and (iii) able to induce apoptosis. It was observed that Cyt c-HA 1MDa has higher stability than its counterpart Cyt c-HA 8kDa, suggesting that the HA molecular weight plays a key role in the formation of a stable bioconjugate. Also, it was noted that A549 cells were more susceptible to the cytotoxic effect of Cyt c than HeLa cells although the cellular uptake of Cyt c-HA was significantly lower as seen in confocal images. This behavior can be understood under the assumption that each cancer cell line has very particular apoptotic and anti-apoptotic mechanisms and overexpression levels of CD44.

Further experiments will be focused on improving the sample construction to avoid random interactions between Cyt c and HA limiting the batch-to-batch reproducibility. Similarly, its stability will be taken into consideration to attain a highly efficient and cytotoxic drug delivery system without the need for a high drug concentration. A smart release approach can be tested to enhance the effectiveness of the system increasing the concentration at target site and decreasing systemic toxicity. To avoid drug resistance, cells can be treated in combination with drugs that alter the expression of anti-apoptotic pathways or proteins making them more susceptible to cytotoxicity from our system; similar experiments have been already performed with success [46].

## Figures and Tables

**Table 1:** Physical characterization of Cyt c-HA bioconjugates.

Samples	Loading %	Zeta potential, mV	Caspase-9 activation %
**Cyt c**	N/A	15.1 ± 0.5	100
Cyt c-HA 8kDa*	35 ± 5	−8.8 ± 0.4	90 ± 6
Cyt c-HA 1MDa*	16 ± 1	−10 ± 1	97 ± 4
**Cyt c-HA 8kDa**	4 ± 1	−11.9 ± 0.7	106 ± 7
**Cyt c-HA 1MDa**	4.3 ± 0.8	−22 ± 1	97 ± 7

* Represent the difference in loading percentage between samples with the same molecular weight HA. According to DLS measurements the size and polydispersity index of Cyt c-HA 8kDa and Cyt c-HA 1MDa was 209.9 nm ± 0.3 and 95.2 nm ± 0.5, respectively.

## Abbreviations

Cyt c: Cytochrome c
HA: Hyaluronic Acid
DDS: Drug Delivery System
EPR: Enhance Permeability and Retention
CD44: Cluster of Differentiation 44
DAPI: 4′,6-Diamidino-2-Phenylindole
PI: Propidium Iodide
FITC: Fluorescein-5-Isothiocyanate
CD: Circular Dichroism
MTS: 3-(4,5-dimethylthiazol-2-yl)-5-(3-carboxymethoxyphenyl)-2-(4-sulfophenyl)-2H-tetrazolium inner salt
MEM: Minimum Essential Medium
DMEM: Dulbecco’s Minimum Essential Medium
FBS: Fetal Bovine Serum
